# Supplementary material for: Metabolite Biomarkers of Leishmania Antimony Resistance
Source: Cells. 2021 Apr 30;10(5):1063. doi: 10.3390/cells10051063 (PMC8146733; doi:10.3390/cells10051063)
Supplement: Supplementary file 1 [file cells-10-01063-s001.zip › cells-1196572-supplementary.pdf]

Supplementary Materials:

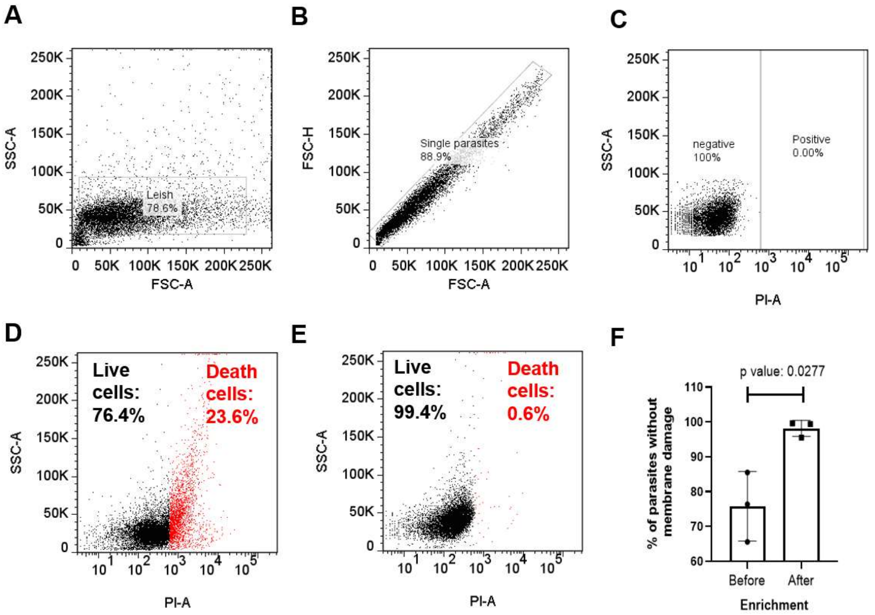

Figure S1. Enrichment of parasites without membrane damage for intracellular metabolomic analysis**.** Cell flow cytometry to estimate the enrichment of parasites without membrane damage. A) Gating Strategy for Flow Cytometry Analysis (A-E). A) *Leishmania*´s population showing heterogeneous size (FSCA-A) and granularity (SSC-A). B) selection of single parasites. C) Fixing the limit to differentiate the incorporating or not of propidium iodide (PI). D) Percentage of parasites without (black dots) and with (red dots) membrane damage before Ficoll gradient centrifugation. E) Percentage of parasites without (black dots) and with (red dots) membrane damage after Ficoll gradient centrifugation. F) Bar plot comparing the percentage of parasites without membrane damage before and after treatment. Ficoll treatment significantly enriched the population of live parasites over 95%. Data normalized by log transformation. Statistical comparison by two tailed unpaired t-student. Degree of freedom: 4. 95% of confidence. Alpha ≤ 0.05.

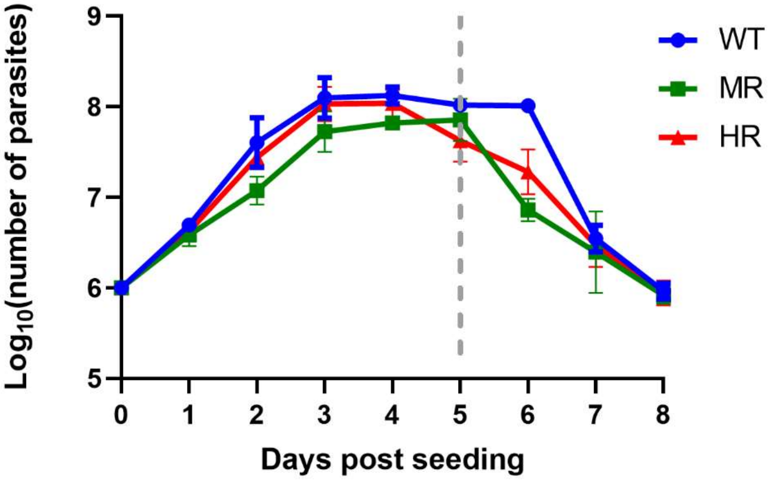

Figure S2. Comparison of the curves of growth between the three *L.tropica* strains. The number of parasites was counted daily using Neubauer chamber. Until the fifth-day post-seeding (stationary phase), the analyzed strains showed comparable curves of growth. WT strain (blue line). MR strain (green line). HR strain (red line). Markers (triangles, boxes or cicles) represent the average of three biological replicates. Error bars show the 95% confidence intervals.

Table S1. Chemical shifts per compound detected in ¹H-NMR spectra of *Leishmania*'s intracellular extracts. Human Metabolome Database (HMDB), PubChem, Kyoto Encyclopedia of Genes and Genomes (KEGG).

ID	Compound Name	HMDB	PubChem	KEGG	Chemical Shifts [± 0.025 ppm]
1	Valine	HMDB0000883	6287	C00183	7.91 (s)
2	Isoleucine	HMDB0000172	6306	C00407	1.33 (dd, J = 6.8, 1.7 Hz, 1H)
3	Propylene glycol	HMDB0001881	1030	C02912	3.24 (s, 1H), 3.7(m); 4.3(m)
4	3-Hydroxyisovalerate	HMDB0000754	69362	C20827	1.25 (s, 1H); 2.38 (s, 1H)
5	Lactate	HMDB0000190	61503	C00186	3.03 (t, J = 7.6 Hz, 1H)
6	Alanine	HMDB0000161	5950	C00041	1.49 (d, J = 7.2 Hz, 5H); 3.82 – 3.77 (m, 1H)
7	Arginine	HMDB0000517	6322	C00062	1.67 (m); 1.73 (m)
8	Acetate	HMDB0000042	176	C00033	1.92(s, 1H)
9	Proline	HMDB0000162	145742	C00148	1.15 (d, J = 6.5 Hz, 1H)
10	Methionine	HMDB0000696	6137	C00073	9.35(s), 9.16(d), 8.84 (d, J = 8.1 Hz, 1H); 8.4(s),6.0(d);8.39(s);6.05 (d, J = 5.8 Hz, 1H)
11	Succinate	HMDB0000254	1110	C00042	1.05 (d, J = 7.1 Hz, 1H); 1.00 (d, J = 7.0 Hz, 1H)
12	Beta-Alanine	HMDB0000043	247	C00719	3.91 (s, 1H), 3.27 (s, 1H)
13	Malate	HMDB0000156	222656	C00149	2.1 (s, 1H)
14	Lysine	HMDB0000182	5962	C00047	2.67 (dd, J = 15.4, 2.9 Hz, 0H)
15	Glycine	HMDB0000157	790	C00262	8.22 (s, 1H), 8.20 (s, 1H)
16	Glycerophosphocholine	HMDB0000086	71920	C00670	2.41 (s, 1H)
17	Betaine	HMDB0002199	10394	C01744	7.19(m), 6.86(m)
18	IMP	HMDB0000175	8582	C00130	1.05 (d, J = 7.0 Hz, 1H)
19	Fumarate	HMDB0000123	750	C00037	3.57 (s, 1)
20	¶-Methylhistidine	HMDB0000001	92105	C01152	7.11 (s, 1H), 7.93 (s, 0H)
21	Desaminotyrosine	HMDB0000142	284	C00058	8.4(s)
22	Xanthine	HMDB0000292	1188	C00385	2.56(t, J= 13.4 Hz, 2H)
23	Hypoxanthine	HMDB0000056	239	C00099	8.6(s), 6.15 (d, J = 5.9 Hz, 1H), 4.5(m)
24	Formate	HMDB0000134	444972	C00122	6.5(s)
25	AMP	HMDB0000045	6083	C00020	8.61(s, 1H); 6.15 (d, J = 5.9 Hz, 1H)
26	NAD+	HMDB0000902	5893	C00003	4.16 – 4.12 (m, 0H), 2.22 – 1.97 (m, 1H)

Table S2. Chemical shifts per compound detected in ^{1}H-NMR spectra of *Leishmania*'s extracellular extracts. Human Metabolome Database (HMDB), PubChem, Kyoto Encyclopedia of Genes and Genomes (KEGG).

ID	Compound Name	HMDB	PubChem	KEGG	Chemical Shifts [± 0.025 ppm]
1	Leucine	HMDB0000687	6106	C00123	0.97 (t, J = 6.2 Hz)
2	Isoleucine	HMDB0000172	6306	C00407	3.70 (d, J = 4.0 Hz), 1.02 (d, J = 7.0 Hz), 0.93(t)
3	Valine	HMDB0000883	6287	C00183	1.00 (d, J = 7.0 Hz, 1H), 1.05 (d, J = 7.0 Hz)
4	Lactate	HMDB0000190	61503	C00186	1.33 (dd, J = 6.8, 1.7 Hz, 1H)
5	Threonine	HMDB0000167	6288	C00188	1.33(d), 4.27(m)
6	Alanine	HMDB0000161	5950	C00041	1.49 (d, J = 7.3 Hz)
7	Lysine	HMDB0000182	5962	C00047	3.03(t),1.7(m), 1.5(m), 1.4(m)
8	Proline	HMDB0000162	145742	C00148	4.14(m)
9	Glutamine	HMDB0000641	5961	C00064	2.46(m), 2.13(m)
10	Succinate	HMDB0000254	1110	C00042	2.4(s)
11	Beta-Alanine	HMDB0000056	239	C00099	2.56(t),3.18(t)
12	Malate	HMDB0000156	222656	C00149	2.67(dd)
13	Aspartate	HMDB0000191	5960	C00049	2.80 (d, J = 4.0 Hz), 2.83 (d, J = 4.0 Hz)
14	Arginine	HMDB0000517	6322	C00062	3.25(t)
15	Glycine	HMDB0000123	750	C00037	3.57(s)
16	L-Serine	HMDB0000187	5951	C00065	3.98(dd), 3.96 (dd)
17	Myoinositol	HMDB0000211	-	C00137	4.28 (qd, J = 6.6, 4.7 Hz)
18	Trehalose	HMDB0000975	7427	C01083	5.21 (d, J = 3.9 Hz, 1H), 3.8-3.89(m)
19	Uracil	HMDB0000300	1174	C00106	5.82 (d, J = 7.7 Hz), 7.56 (d, J = 7.7 Hz)
20	Fumarate	HMDB0000134	444972	C00122	6.52(s)
21	N-acetyl tyrosine	HMDB0000866	68310	C01657	7.16(d), 6.88(d)
22	Tyrosine	HMDB0000158	6057	C00082	6.9(d),7.2(d)
23	Phenylalanine	HMDB0000159	6140	C00079	7.44(t),7.39(t),7.35(d)
24	Tryptophan	HMDB0000929	6305	C00078	7.54(d),7.72(d)
25	¶-Methylhistidine	HMDB0000001	92105	C01152	7.98 (s), 7.17(s)
26	Hypoxanthine	HMDB0000157	790	C00262	8.22(s), 8.24(s)
27	Formate	HMDB0000142	284	C00058	8.47(s)
28	Imidazole	HMDB0001525	795	C01589	8.36(s)
29	Nicotinate	HMDB0001488	938	C00253	8.95(d), 8.64(dd)

Table S3. Raw data from Venn diagram comparing the different and common compounds detected at the intracellular and extracellular level in *Leishmania* parasites by ^{1}H-NMR. Three groups are shown: "extracellular" representing the compounds exclusively detected at the extracellular level, "intracellular" representing the compounds exclusively detected at the intracellular level, and "extracellular and intracellular" representing the group of compounds detected in both approaches.

Group	Total	Compound	HMDB	PubChem	KEGG
Extracellular and Intracellular	15	L-Isoleucine	HMDB0000172	6306	C00407
		L-Arginine	HMDB0000517	6322	C00062
		L-Valine	HMDB0000883	6287	C00183
		Glycine	HMDB0000123	750	C00037
		Formic acid	HMDB0000142	284	C00058
		L-Lysine	HMDB0000182	5962	C00047
		L-Alanine	HMDB0000161	5950	C00041
		Succinic acid	HMDB0000254	1110	C00042
		Beta-Alanine	HMDB0000056	239	C00099
		L-Proline	HMDB0000162	145742	C00148
		1-Methylhistidine	HMDB0000001	92105	C01152
		Fumaric acid	HMDB0000134	444972	C00122
		Hypoxanthine	HMDB0000157	790	C00262
		L-Lactic acid	HMDB0000190	61503	C00186
		L-Malic acid	HMDB0000156	222656	C00149
Intracellular (only)	11	Inosinic acid	HMDB0000175	8582	C00130
		Xanthine	HMDB0000292	1188	C00385
		Acetic acid	HMDB0000042	176	C00033
		Propylene glycol	HMDB0001881	1030	C02912
		Desaminotyrosine	HMDB0002199	10394	C01744
		Adenosine monophosphate	HMDB0000045	6083	C00020
		Glycerophosphocholine	HMDB0000086	71920	C00670
		L-Methionine	HMDB0000696	6137	C00073
		Betaine	HMDB0000043	247	C00719
		3-Hydroxyisovaleric acid	HMDB0000754	69362	C20827
		NAD	HMDB0000902	5893	C00003
Extracellular (only)	14	L-Tryptophan	HMDB0000929	6305	C00078
		Trehalose	HMDB0000975	7427	C01083
		L-Leucine	HMDB0000687	6106	C00123
		N-Acetyl-L-tyrosine	HMDB0000866	68310	C01657
		L-Tyrosine	HMDB0000158	6057	C00082
		L-Threonine	HMDB0000167	6288	C00188
		Nicotinic acid	HMDB0001488	938	C00253
		myo-Inositol	HMDB0000211	-	C00137
		L-Glutamine	HMDB0000641	5961	C00064
		Imidazole	HMDB0001525	795	C01589
		L-Aspartic acid	HMDB0000191	5960	C00049
		Uracil	HMDB0000300	1174	C00106
		L-Serine	HMDB0000187	5951	C00065
		L-Phenylalanine	HMDB0000159	6140	C00079

Table S4. Total compounds detected by ^{1}H-NMR in *L. tropica* distributed by the metabolites main class.

Metabolites main class	Total	Expected	Hits	Raw p	Holm p	FDR
Amino acids	723	0.146	18	$1.09X10^{-33}$	$2.65X10^{-31}$	$2.65X10^{-31}$
TCA acids	9	0.001	3	$6.36X10^{-10}$	$1.55X10^{-7}$	$7.77X10^{-8}$
Purines	89	0.017	2	$1.53X10^{-4}$	0.037	0.012
Disaccharides	9	0.001	1	0.001	0.437	0.111

Table S5. Total compounds distributed by KEGG metabolic pathway. Expected, number of compounds expected by chance; hits, number of detected compounds matching with each metabolic pathway; Raw p, raw p-value; Holm p, adjusted p-value by Holm-Bonferroni method; FDR, false discovery rate.

KEGG pathway	Total	Expected	Hits	Raw p	Holm p	FDR
Aminoacyl-tRNA biosynthesis	48	1.26	16	$2.38X10^{-15}$	$2X10^{-13}$	$2X10^{-13}$
Valine, leucine, and isoleucine biosynthesis	8	0.21	4	$2.66X10^{-5}$	$2.21X10^{-3}$	$1.12X10^{-3}$
Arginine biosynthesis	14	0.368	4	$3.39X10^{-4}$	0.028	$9.5X10^{-3}$
Alanine, aspartate, and glutamate metabolism	28	0.736	5	$6.12X10^{-4}$	0.049	0.013
Glyoxylate and dicarboxylate metabolism	32	0.841	5	$1.16X10^{-3}$	0.093	0.017
Pantothenate and CoA biosynthesis	19	0.499	4	$1.19X10^{-3}$	0.094	0.017
Phenylalanine, tyrosine, and tryptophan biosynthesis	4	0.105	2	3.9110^{-3}	0.305	0.047
Nicotinate and nicotinamide metabolism	15	0.394	3	$6.15X10^{-3}$	0.474	0.064

Table S6. Comparison of statistical measures calculated for the supervised OPLS-DA models for the differentiation of Sb^{III}-sensitive and resistant parasites at the intracellular and extracellular levels. Explained variation (R2Y). Predictive ability of the model (Q2). Total number of samples/observation (N).

**Data**	**Outliers**	**OPLS-DA scores**
Intracellular metabolic profiling dataset	Outliers included	R2Y= 0.978<br>Q2= 0.997<br>N= 9
Extracellular metabolic profiling	Outliers included	R2Y=0.946<br>Q2=0.844<br>N=26

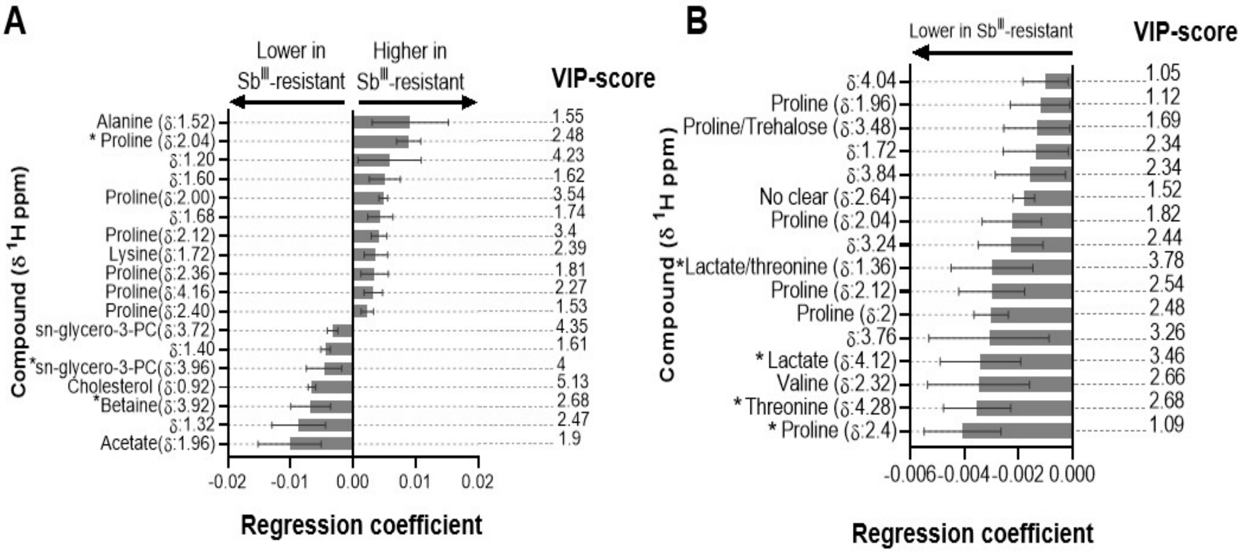

Figure S3. Regression coefficients and VIP scores based on the metabolomic profiling dataset for the OPLS-DA models comparing Sb^{III} resistant parasites versus Sb^{III} sensitive parasites. Chemical shift (δ) and or assigned metabolites are represented in the Y-axis, while the regression coefficient is plotted in the X-axis. A) Intracellular extracts. B) Extracellular extracts. Positive values of coefficients (right-facing bars) indicate increased metabolite in Sb^{III} resistant parasites (fold change>1) while negative values (left-facing bars) represent a decrease in metabolite concentrations (fold change<1). Only significant metabolite/protein-mediator are shown ($p<0.05$; jackknife technique).

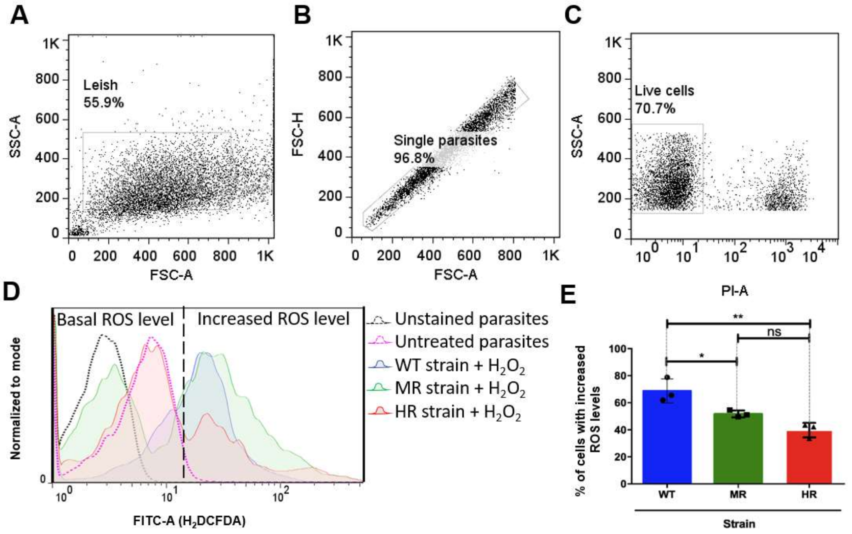

Figure S4. Sb^{III} resistant parasites show better tolerance to oxidative stress induced by hydrogen peroxide**.** Gating Strategy for Flow Cytometry Analysis (A-D). A) Leishmania´s population showing heterogeneous size (FSCA-A) and granularity (SSC-A). B) selection of single parasites. C) Selection of parasites without membrane damage or propidium iodide (PI) incorporation. D) Intracellular ROS levels quantified as the incorporation of the H2DCFDA probe. The basal level of ROS was fixed based on untreated parasites or control group. Under H_2O_2 treatment, SbIII-sensitive (WT) parasites were mostly distributed in the region of parasites with high ROS levels (right panel), high resistant (HR) parasites, were mostly distributed in the region of lower ROS levels (left panel), while moderately resistant (MR) parasites were almost equally distributed between two panels. E) Statistical analysis comparing the percentage of parasites with increased ROS level per experimental condition. The bars represent the averaged cell number producing higher ROS levels under H_2O_2 exposure. Three independent biological replicates were done in each experimental condition. WT strain (blue bar). MR strain (green bar). HR strain (red bar). Statistical analysis included ANOVA one way followed by Tukey´s multiple comparisons test. p-value > 0.05 (ns), p-value ≤ 0.05 (*), ≤ 0.01 (**). Forward scatter area (FSC-A). Forward scatter height (FSC-H). Side scatter area SSC-A.
